# Adding Bismuth to Rabeprazole-Based First-Line Triple Therapy Does Not Improve the Eradication of *Helicobacter pylori*

**DOI:** 10.1155/2017/5320180

**Published:** 2017-07-16

**Authors:** Meng-Chieh Wu, Yao-Kuang Wang, Chung-Jung Liu, Fang-Jung Yu, Fu-Chen Kuo, Min-Li Liu, Chao-Hung Kuo, Deng-Chyang Wu, Yao-Kang Huang, I-Chen Wu

**Affiliations:** ^1^Division of Gastroenterology, Department of Internal Medicine, Kaohsiung Medical University Hospital, Kaohsiung, Taiwan; ^2^Department of Internal Medicine, Kaohsiung Municipal Ta-Tung Hospital, Kaohsiung, Taiwan; ^3^Faculty of Medicine, Department of Medicine, College of Medicine, Kaohsiung Medical University, Kaohsiung, Taiwan; ^4^School of Medicine, College of Medicine, E-Da Hospital, I-Shou University, Kaohsiung, Taiwan; ^5^Department of Pharmacy, E-Da Cancer Hospital, Kaohsiung, Taiwan; ^6^Division of Gastroenterology, Department of Internal Medicine, Ten-Chan General Hospital, Chung-Li, Taoyuan, Taiwan

## Abstract

This randomized controlled study aimed to evaluate whether adding bismuth to the standard first-line triple therapy could improve the eradication rate of *Helicobacter pylori*. A total of 162 patients with *Helicobacter pylori* infection were randomly assigned to either the 7-day triple therapy group (RAK regimen: rabeprazole 20 mg, amoxicillin 1 g, and clarithromycin 500 mg bid; *n* = 81) or the bismuth plus triple therapy group (*n* = 81). In the RBAK group, bismuth subcitrate 360 mg twice daily was added to the RAK regimen. A follow-up endoscopy or urea breath test was performed at least 4 weeks after eradication to confirm the treatment efficacy. Comparable compliance and *Helicobacter pylori* eradication rates were observed in both groups in either intention-to-treat [RAK 72.8% (59/81) versus RBAK 77.8% (63/81); *p* = 0.47] or per protocol analysis [RAK 74.7% (59/79) versus RBAK 81.8% (63/77); *p* = 0.26]. Adverse effects were commonly reported (50.6% for both groups) although most of these did not cause cessation of treatment. The resistance rate was 27.2% for metronidazole and 12.3% for clarithromycin. Adding bismuth to the standard 7-day triple therapy did not substantially increase the eradication rate. Further study is needed clarifying whether extending the duration of RBAK regimen to 10–14 days can lead to a better result.

## 1. Introduction

*Helicobacter pylori (H. pylori)* is an important cause of gastritis, peptic ulcer, and gastric cancer [1]. In 1997, the Maastricht Consensus Meeting suggested standard triple therapy including proton pump inhibitor (PPI), clarithromycin, and amoxicillin given twice daily for 7 days as the first-line treatment for *H. pylori* with treatment regimens achieving an eradication rate of over 80% [2]. However, with increasing antibiotic resistance and decreasing efficacy of the standard triple therapy globally, identification of a better first-line regimen has become an essential issue. A higher clarithromycin resistance rate results in lower treatment efficacy, which falls below 85% for standard 7-day triple therapy if the prevalence of clarithromycin resistance is 15% [3]. In 2010, Graham and colleagues indicated that the eradication rate of triple therapy was >80% in only 40% of studies reviewed and >85% in 18% of such studies [4]. Maastricht V Consensus also suggested avoiding standard triple therapy as first-line regimen in areas of high clarithromycin resistance (>15%) [5].

Bismuth-containing therapy is one of the proposed substitutes to replace standard triple therapy [5]. Bismuth exerts its antimicrobial effect by suppressing *H. pylori*, although it is unable to eliminate the organism [6]. In addition to clarithromycin, there is an increasing resistance to metronidazole and levofloxacin in many countries [7]. In Taiwan, the clarithromycin resistance rate increased from 10.1% in 2000–2007 to 12.8% in 2011-2012 [8]. Recent studies reported variable eradication rates (77.3–81.6% in intention-to-treat [ITT] analysis) using standard 7-day triple therapy in Taiwan [9, 10]. In the World Gastroenterology Organization Global Guidelines, bismuth-containing quadruple therapy including PPI-bismuth plus two antibiotics, either tetracycline and metronidazole or amoxicillin and clarithromycin, is recommended as one of the first-line treatments in developing countries [11]. A study in Thailand showed 92% eradication rate via adding bismuth to the standard 7-day triple therapy [12]. However, a poor eradication efficacy (~68%) using such an approach was reported in China [13]. Since the clarithromycin resistance is still less than 15% in Taiwan, we aimed to investigate whether adding bismuth to 7-day rabeprazole-based standard triple therapy can increase the eradication efficacy.

## 2. Patients and Methods

### 2.1. Patients

From April 2013 to January 2015, patients who visited the Gastroenterology Clinic of Ten Chan Hospital with complaints of dyspepsia or epigastric discomfort were invited to participate in this study. All of them were aged over 20 years and received upper gastrointestinal endoscopy to confirm the diagnosis and positive *H. pylori* infection. Those who had taken antibiotics, bismuth, or proton pump inhibitors within the previous 4 weeks were excluded. Other exclusion criteria included allergy to any drug in the study, prior gastric surgery, severe concomitant diseases (e.g., decompensate liver cirrhosis, uremia, and gastric cancer), previous *H. pylori* eradication therapy, pregnancy, or lactation.

### 2.2. Study Design

**Figure 1:**
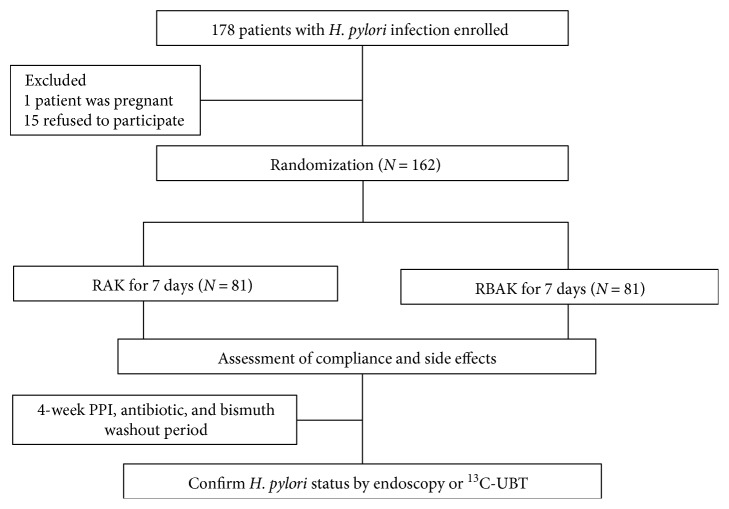
Flowchart.

The study flow chart is shown in Figure 1. All participants underwent upper endoscopic examinations; biopsy samples were taken from gastric body and antrum to detect *H. pylori* by rapid urease test, histology, and culture. The presence of *H. pylori* was defined as (i) a positive result of culture or (ii) positive results of both rapid urease test and histology. The participants were interviewed by trained study nurses using a standardized questionnaire to obtain their demographic data and medical history.

Once confirmed with *H. pylori* infection and having signed the informed consent, participants were randomly assigned to either the standard triple-therapy group (RAK: rabeprazole 20 mg, amoxicillin 1 g, and clarithromycin 500 mg, all twice daily for 7 days) or the bismuth plus standard triple-therapy group (RBAK: rabeprazole 20 mg, bismuth subcitrate 360 mg, amoxicillin 1 g, and clarithromycin 500 mg, all twice daily for 7 days). A computer-generated random number was chosen for randomization. The patients and physicians were not blinded to the therapy assigned.

After completion of *H. pylori* eradication therapy, participants were asked to come back for collection of the information on any adverse event of drug compliance. In order to avoid false negative results, they were also asked to have a 4-week PPI, antibiotic, and bismuth washout period before further examination of *H. pylori* status. The second endoscopy with rapid urea test, histology, and culture or ^13^C-urea breath test (UBT) for those who refused endoscopic exams was carried out at the end of the washout period. Those who did not return to confirm their *H. pylori* status were deemed as treatment failure in intention-to-treat (ITT) analysis. This study was approved by the Institutional Review Board of Kaohsiung Medical University Hospital (KMUH-IRB-20120028), Kaohsiung, Taiwan. This study has been registered to the ClinicalTrials.gov Protocol Registration and Result System (PRS); the registry number was NCT03108287.

### 2.3. Questionnaire

The questionnaire used here covered the participants' personal and medical history, adverse effects, and compliances. Smokers were defined as those who smoked more than one pack of cigarettes per week. Alcohol drinkers were defined as those who had at least one cup of alcoholic beverage 3 days a week. The adverse effects included abdominal pain, diarrhea, constipation, headache, poor appetite, nausea/vomiting, skin rash, dizziness, taste change, and others. The participants who deemed these newly onset symptoms disturbing were recognized as having major adverse effects, and those who did not consider the symptoms as constituting a disturbance to their daily life were defined as having minor adverse effects. Compliance was evaluated by counting the remaining medication at the end of treatment. Poor compliance was defined as consumption of less than 70% of the prescription medication.

### 2.4. Diagnosis of *H. pylori* Infection

#### 2.4.1. Pathological Examination and Culture

Biopsy specimens taken from the lesser curvature of the gastric antrum and angularis were sent for pathology with hematoxylin and eosin stain and bacterial cultures. The pathologists read the results and diagnosed the severity of gastritis according to the updated Sydney classification. For culture, the tissues were rubbed on a Columbia blood agar plate and incubated at 35°C under microaerobic conditions for 4-5 days. A positive culture of *H. pylori* was considered in the presence of Gram-negative, oxidase, catalase, and urease-positive colonies.

#### 2.4.2. Rapid Urease Test

The detailed method has been described previously [14]. In brief, the Campylobacter-like organism test (Delta West, Perth, WA, Australia) was used and the results were obtained 24 hours after examination.

#### 2.4.3. ^13^C-Urea Breath Test (UBT)

The *^13^*C-urea was manufactured by the Institute of Nuclear Energy Research in Taiwan, and the protocol was described in the previous report [14]. One hundred mg of 99% ^13^C-labeled urea was dissolved in 50 ml sterile water. The expired air was collected before and 30 min after the patients drank the water containing ^13^C-labeled urea, and the samples were analyzed. The cut-off value for a positive result was an increase of 4 per mil of ^13^CO_2_ in the 30 min sample subtracting that in the baseline sample. The technician who performed UBT and read the results was blinded to the regimen assigned.

#### 2.4.4. Antimicrobial Resistance

After *H. pylori* was successfully cultured, it was sent for examination of antibiotic resistance. The detailed methods and criteria chosen as the minimal inhibitory concentration were based on the previous report [15]. A Campy-blood agar plate [Brucella agar (Difco, Detroit, MI, USA) + Iso-Vitalex (Gibco, Grand Island, NY, USA) + 10% whole sheep blood] was used to isolate resistance strains of *H. pylori*. Epsilometer test (E-test: AB Biodisck, Solna, Sweden) was used to test antibiotic resistance against clarithromycin, tetracycline, metronidazole, amoxicillin, and levofloxacin. *H. pylori* strains with minimal inhibitory concentrations >1, >4, >8, >0.5, and >1 mg/L were considered to be resistant to clarithromycin, tetracycline, metronidazole, amoxicillin, and levofloxacin, respectively.

#### 2.4.5. Statistical Analysis

All the data was analyzed using Stat View 5.0.1 (SAS Institute, Cary, NC, USA). A *χ*^2^ test and Student's *t*-test were used in this study. It was evaluated by ITT and per-protocol (PP) analysis as the major outcome for the rate of successful *H. pylori* eradication. Independent factors were evaluated by univariate analysis and confirmed by logistic regression. A *p* value <0.05 was considered statistically significant. Assuming that the conventional eradication rate (RAK group) is 77% [9], and the RBAK group was to achieve 85% eradication rate, an 8% difference of increase, our statistical power would be 85% under the sample sizes of about 198 subjects in each group and two-sided *p* value of 0.05 if 95% of patients (*N* = 188) completed the follow-up [16].

## 3. Results

This study was terminated early because the interim analysis showed a lower efficacy of the RBAK regimen (~78% in ITT analysis) than we expected. In total, 178 *H. pylori*-infected patients were enrolled and 16 were excluded due to pregnancy or refusal to participate (Figure 1). Eighty-one of the remaining 162 participants were randomized into the RAK group, while the other half was placed in the RBAK group. There were no significant differences in age, gender, smoking and alcohol consumption, anticoagulant, nonsteroidal anti-inflammatory drugs (NSAIDs), and prednisolone use in both groups (Table 1). In ITT analysis, the eradication rates was 72.8% (59/81) in the RAK group and 77.8% (63/81) in the RBAK group (*p* = 0.47, Table 2). PP analysis was 74.7% (59/79) in the RAK group and 81.8% (63/77) in the RBAK group (*p* = 0.28). Six patients took less than 70% of the total medication and were deemed to have poor compliance. The compliance was 97.5% in the RAK group and 95.1% in the RBAK group (*p* = 0.40, Table 2). Similar prevalence of overall (both 50.6%, Table 2) and detailed side effects (Table 3) was observed. The most common side effects were bad taste, followed by diarrhea, and abdominal pain. All of the events subsided spontaneously or were medically manageable.

*H. pylori* strains were successfully isolated from 50% (81/162) of the participants (Table 4). The overall antimicrobial resistance rates were 27.2% for metronidazole, 12.3% for clarithromycin, 0% for tetracycline, 11.1% for levofloxacin, and 2.5% for amoxicillin (Table 4). The prevalence of antibiotic resistance was similar in both groups. Clinical factors, including antibiotic resistance, presence of dual resistance, adverse effects, compliance, and smoking, that might influence the eradication rate of the two regimens were examined (Table 5). Although not statistically significant, presence of clarithromycin resistance did result in a very low eradication efficacy in both groups (40% versus 20%, *p* = 1.00). Dual resistance, defined as resistance to any two of the five antibiotics in the table, was also related to a lower success rate in *H. pylori* treatment (ITT: 60% in both groups, Table 5).

## 4. Discussion

Our results showed slightly higher but not satisfactory eradication results by adding bismuth to the standard 7-day rabeprazole-based triple therapy in Northern Taiwan. A recent study in Taiwan reported an 83.7% eradication rate using PPI-amoxicillin-clarithromycin for 14 days [17]. The bismuth quadruple therapy (bismuth dicitrate 300 mg qid, lansoprazole 30 mg bid, tetracycline 500 mg qid, and metronidazole 500 mg tid for 10 days) appeared to be a better first-line treatment (90.4% by ITT analysis) [17]. However, their clarithromycin resistance rate was higher (14–17%) than our study (12.3%). In Thailand, there was a high eradication rate (92–100%) using 7- or 14-day triple therapy (lansoprazole, amoxicillin, and clarithromycin) plus bismuth with or without probiotics [12]. However, the sample size was small (25 cases in each group) and their clarithromycin resistance rate was very low (<4%). Another study in mainland China did not find adding bismuth to the 10-day triple therapy an effective regimen (68.4% versus 68% by ITT analysis) [18]. Different antibiotic resistance rates across areas could be an important cause for such discrepancies.

According to Maastricht V Consensus, bismuth-containing quadruple treatments are the recommended first-line therapy in areas of high clarithromycin resistance [5]. It was also suggested that PPI-clarithromycin-containing triple therapy should be abandoned in areas with clarithromycin resistance rate over 15% (low evidence level, strongly recommended) [5]. The clarithromycin resistance rate was 12.3% in the present study. However, the eradication rate was only 72.8% by ITT analysis for 7-day triple therapy; adding bismuth increased the success rate by 5%. Further extending the duration of RBAK regimen to 10–14 days or substitute clarithromycin with another antibiotic may be a better strategy. In a meta-analysis, 10-day bismuth quadruple therapy had better efficacy than 7-day standard triple therapy while there were similar compliance and side effect rates [19]. Ciccaglione et al. indicated that adding bismuth to pantoprazole, amoxicillin and moxifloxacin had achieved a significantly higher eradication rate (92% versus 77.1%, *p* < 0.03) [20]. In another study, the moxifloxacin-tetracycline-lansoprazole regimen plus bismuth was a better regimen than that without bismuth (82.1% versus 55.4%) [21]. Because bismuth citrates inhibit the motility of *H. pylori,* cause morphological destruction, and suppress the growth of *H. pylori*, it can be a component in *H. pylori* eradication regimen [22].

With the increasing antibiotic usage, drug resistance has become a global issue and substantially influences the efficacy of *H. pylori* eradication therapy [23]. In Europe, the drug resistance rate was 17.5% for clarithromycin, 34.9% for metronidazole, and the rate of clarithromycin resistance was different across European countries [24]. Resistance to clarithromycin, metronidazole, and fluoroquinolone is also increasing in China [13]. In Taiwan, the clarithromycin resistance rate in our study was very similar to some reports (11.4–12.8%) [8, 25], but lower than that (14–17%) in a recent large-scale study [17]. This suggested that local clarithromycin resistance, even in the same country, is a key factor when choosing the most appropriate first-line treatment for *H. pylori*.

In conclusion, with the increasing clarithromycin resistance in Taiwan, 7-day triple therapy may no longer be a suitable first-line therapy even after adding bismuth. Further study is needed to clarify whether extending the duration of RBAK regimen to 10–14 days can lead to a better result.

## Figures and Tables

**Table 1:** Characteristics of RAK and RBAK therapies.

	RAK	RBAK	*p* value
Patients (*N*)	81	81	
Male/female (*n*)	35/46	40/41	0.43
Age (years, mean ± SD)	49.80 ± 14.4	51.16 ± 12.5	0.26
NSAIDs, *n*/*N* (%)	6/81 (7.4)	6/81 (7.4)	—
Prednisolone use, *n*/*N* (%)	1/81 (1.2)	1/81 (1.2)	—
Anticoagulant, *n*/*N* (%)	0/81 (0)	0/81 (0)	—
Smoke, *n*/*N* (%)	23/81 (28.4)	20/81 (24.7)	0.60
Alcohol, *n*/*N* (%)	10/81 (12.3)	6/81 (7.4)	0.29
Gastritis, *n*/*N* (%)	68/81 (84.0)	72/81 (88.9)	0.36
GU, *n*/*N* (%)	16/81 (19.8)	17/81 (21.0)	0.84
DU, *n*/*N* (%)	32/81 (39.5)	35/81 (43.2)	0.63
GU + DU, *n*/*N* (%)	4/81 (4.9)	12/81 (14.8)	0.04

**Table 2:** Major outcomes of RAK and BRAK for *Helicobacter pylori* eradication.

	RAK	BRAK	*p* value
Eradication rate, *n*/*N* (%)			
Intention-to-treat	59/81 (72.8)	63/81 (77.8)	0.47
Per protocol	59/79 (74.7)	63/77 (81.8)	0.28
Compliance, *n*/*N* (%)	79/81 (97.5)	77/81 (95.1)	0.40
Side effect, *n*/*N* (%)	41/81 (50.6)	41/81 (50.6)	—

7 patients had an unknown *H. pylori* status after treatment (3 in RAK group, 4 in RBAK group).

**Table 3:** Adverse events of RAK and RBAK therapies for *Helicobacter pylori* eradication.

Adverse events, *n* (%)	RAK (*N* = 81)	RBAK (*N* = 81)	*p* value
Abdominal pain	9 (11.1)	12 (14.8)	0.48
Diarrhea	14 (17.3)	11 (13.6)	0.52
Constipation	3 (3.7)	4 (4.9)	0.70
Headache	3 (3.7)	1 (1.2)	0.31
Anorexia	1 (1.2)	1 (1.2)	—
Nausea	3 (3.7)	2 (2.5)	0.65
Skin rash	2 (2.5)	1 (1.2)	0.56
Dizziness	3 (3.7)	2 (2.5)	0.65
Bad taste	25 (30.9)	20 (24.7)	0.38
Fatigue	9 (11.1)	7 (8.6)	0.60
Others	7 (8.6)	15 (18.5)	0.07
Overall	41 (50.6)	41 (50.6)	—

**Table 4:** Rate of antimicrobial resistance of *Helicobacter pylori.*

Antimicrobial resistance, *n* (%)	RAK (*N* = 38)	BRAK (*N* = 43)	*p* value	Total (*N* = 81)
Etronidazole	11 (28.9)	11 (25.6)	0.73	22 (27.2)
Clarithromycin	5 (13.2)	5 (11.6)	0.83	10 (12.3)
Tetracycline	0 (0.0)	0 (0.0)	—	0 (0.0)
Levofloxacin	3 (7.9)	6 (14.0)	0.38	9 (11.1)
Amoxicillin	1 (2.6)	1 (2.3)	0.93	2 (2.5)

**Table 5:** Univariate analysis of clinical factors which might influence the efficacy of the RAK and RBAK.

Eradication rate, *n*/*N* (%)	RAK	RBAK	*p* value
Resistance to			
Metronidazole^∗^			
ITT and PP	6/11 (54.5)	7/11 (63.6)	1.00
Clarithromycin^∗^			
ITT and PP	2/5 (40.0)	1/5 (20.0)	1.00
Tetracycline	—	—	—
Levofloxacin^∗^	3/3 (100)	3/6 (50.0)	0.46
Amoxicillin^∗^	1/1 (100)	1/1 (100)	1.00
Dual resistance^¶^			
Present^∗^			
ITT	3/5 (60.0)	3/5 (60.0)	1.00
PP	3/5 (60.0)	3/4 (75.0)	1.00
Absent			
ITT	27/33 (81.8)	27/38 (71.1)	0.29
PP	27/31 (87.1)	27/35 (77.1)	0.30
Adverse events			
Present	25/36 (69.4)	34/41 (82.9)	0.16
Absent	31/40 (77.5)	29/40 (72.5)	0.61
Compliance			
Good	58/80 (72.5)	63/81 (77.8)	0.44
Poor^∗^	1/1 (100)	0/0 (0.0)	—
Smoking			
Present	17/23 (73.9)	15/20 (75.0)	0.94
Absent	42/58 (72.4)	48/61 (78.7)	0.42

^∗^Fisher exact test. ^¶^Dual resistance was defined as presence of any two of the five antibiotics.
